# The type IV pilus assembly ATPase PilB functions as a signaling protein to regulate exopolysaccharide production in *Myxococcus xanthus*

**DOI:** 10.1038/s41598-017-07594-x

**Published:** 2017-08-04

**Authors:** Wesley P. Black, Lingling Wang, Xing Jing, Rafael Castañeda Saldaña, Feng Li, Birgit E. Scharf, Florian D. Schubot, Zhaomin Yang

**Affiliations:** Department of Biological Sciences, Virginia Polytechnic Institute and State University, Blacksburg, VA 24061 USA

## Abstract

*Myxococcus xanthus* possesses a form of surface motility powered by the retraction of the type IV pilus (T4P). Additionally, exopolysaccharide (EPS), the major constituent of bacterial biofilms, is required for this T4P-mediated motility in *M. xanthus* as the putative trigger of T4P retraction. The results here demonstrate that the T4P assembly ATPase PilB functions as an intermediary in the EPS regulatory pathway composed of the T4P upstream of the Dif signaling proteins in *M. xanthus*. A suppressor screen isolated a *pilB* mutation that restored EPS production to a T4P^−^ mutant. An additional PilB mutant variant, which is deficient in ATP hydrolysis and T4P assembly, supports EPS production without the T4P, indicating PilB can regulate EPS production independently of its function in T4P assembly. Further analysis confirms that PilB functions downstream of the T4P filament but upstream of the Dif proteins. *In vitro* studies suggest that the nucleotide-free form of PilB assumes the active signaling conformation in EPS regulation. Since *M. xanthus* PilB possesses conserved motifs with high affinity for c-di-GMP binding, the findings here suggest that c-di-GMP can regulate both motility and biofilm formation through a single effector in this surface-motile bacterium.

## Introduction

*Myxococcus xanthus* is a motile bacterium well adapted to life on a solid surface. While a unicellular organism, it possesses a social or multicellular life style during both its vegetative and developmental phases^1, 2^. During vegetative growth, groups of *M. xanthus* cells swarm over solid surfaces to digest other bacteria and organic macromolecules as nutrients^3^. Its developmental program, which is triggered by nutrient limitation, involves the aggregation of ~10^5^ cells on surfaces by directed cell movement^4, 5^; these multicellular aggregates eventually mature into fruiting bodies when vegetative cells within cease to move and differentiate into dormant spores embedded in an exopolysaccharide (EPS) matrix^6, 7^. When environmental conditions become conducive for growth, spores can germinate to resume proliferation and swarming. Motility is critical to this bacterium because both vegetative predation and developmental aggregation require cells to move on solid surfaces.

The surface motility of *M. xanthus* entails social (S) and adventurous (A) gliding systems^4, 8^. Mutations inactivating one system rarely affect the other, indicating the existence of two sets of motility machineries^9–11^. Phenotypically, A motility is functional even when cells are well isolated, whereas S motility requires cells to be in close proximity or in groups^9^. There is evidence that traveling motor complexes within cells drive A motility^12–15^. The motor for S motility is the type IV pilus (T4P)^16–19^. The T4P protein filament, which mostly localizes to one cell pole in *M. xanthus* at a given time^20, 21^, is also known to power the related twitching motility in other bacteria^22, 23^. The distal end of a T4P first attaches to an anchor and its ensuing retraction at the proximal end pulls the cell forward^24, 25^. In *M. xanthus*, the preferred anchor for T4P retraction is EPS either associated with the cell surface or deposited on a solid substratum by this bacterium^26, 27^. This preference explains the “social” aspect of T4P-mediated motility in *M. xanthus* because a cell requires its EPS-bearing or EPS-depositing neighbors to anchor and activate the retraction of its T4P motor. EPS, which is a key constituent of the matrix for biofilms and for the organization of *M. xanthus* fruiting bodies^6, 7^, has been shown recently to affect *M. xanthus* motility behavior independently of its role as anchors for T4P retraction in S motility^28^.

The level of EPS in *M. xanthus* is regulated by a signal transduction pathway consisting of the Dif chemosensory proteins as well as the T4P machinery (T4PM)^24, 29–31^. DifA, DifC and DifE resemble the methyl-accepting chemoreceptor proteins (MCPs), the scaffold CheW, and the histidine kinase CheA, respectively^27^. These three proteins form a transmembrane (TM) signaling complex that positively regulates EPS production through the kinase DifE^32, 33^. DifD, a CheY-like substrate of DifE phosphorylation, functions as a phosphate sink to negatively regulate EPS production^29, 32^. DifG, a homologue of the CheC phosphatase, is a negative regulator that can dephosphorylate DifD-phosphate^29, 32^. The occurrence of T4P correlates closely with that of EPS^31^. T4P^−^ mutants are EPS^-^ whereas the hyperpiliated *pilT* mutant is EPS^+^. Since mutations in *dif* are epistatic to those in T4P or *pil* genes, T4P has been proposed to function as a sensory apparatus that perceives and transmits signals to the Dif proteins downstream. The communication between T4P and Dif is mediated in part by the negative regulator StkA, a DnaK-like protein^34, 35^ that acts downstream of T4P but upstream of Dif^36^. Many questions remain concerning the mechanism of EPS regulation by this pathway although the transcription of *eps* genes does not appear to be the target of this regulation^37^.

We demonstrate here that the *M. xanthus* T4P assembly ATPase PilB^21^ functions in a regulatory capacity in signaling EPS production independently of T4P assembly. Genetic studies uncovered that mutations in *pilB* can suppress the EPS defects resulting from the deletion of the pilin gene *pilA*. A mutation known to eliminate the ATPase activity of PilB and its ability to support T4P assembly^21^ was found to strongly suppress the EPS defect of the *pilA* deletion strain. This observation indicates that the role of PilB in EPS regulation can be independent of its role as the T4P assembly ATPase. Analysis *in vitro* suggests that it is the nucleotide-free or the apo form of PilB that actively signals EPS production. Our results support the conclusion that PilB functions in a signaling capacity with dual roles in the regulation of motility and biofilm formation in *M. xanthus*.

## Results

### Isolation of *pil* mutations suppressing the EPS^−^ phenotype of a *pilA* deletion

To understand how the T4P filament functions to regulate EPS in *M. xanthus*, the *pil* genes encoding the T4P structural proteins were targeted in a genetic screen for suppressors of a *pilA* deletion (Δ*pilA*)^17, 38^. For this suppressor screen, 11 genes in three clusters at the *M. xanthus pil* locus (Fig. S1) were mutagenized. These are the *pilB*, *pilT* and *pilC* (*pilBTC*) genes in one cluster, *pilG*, *pilH* and *pilI* (*pilGHI*) as well as *pilM*, *pilN*, *pilO*, *pilP* and *pilQ* (*pilMNOPQ*) in two other clusters. We first deleted *pilBTC*, *pilGHI* and *pilMNOPQ* as individual clusters in the WT and the Δ*pilA* backgrounds. These mutants were confirmed to be defective in both S motility and EPS production (Fig. S2)^31^. Next, three complementation plasmids containing *pilBTC*, *pilGHI* and *pilMNOPQ* were constructed. These plasmids, which can integrate into the *M. xanthus* chromosome at a phage attachment site^39^, were transformed into and demonstrated to complement its corresponding deletions in S motility and EPS production in the WT background (Fig. S2). Transforming these plasmids into their respective deletion mutants in the Δ*pilA* background resulted in strains without S motility and EPS (Fig. S2) as expected for a Δ*pilA* strain^31^.

The three plasmids were mutagenized in an *Escherichia coli* mutator strain^40^, and pools of mutagenized plasmids were isolated and transformed into their respective deletion strains in a Δ*pilA* background for the suppressor screen. Approximately 20,000 transformants for each plasmid were screened on plates with Congo red where EPS^+^ colonies appear red but EPS^−^ ones are unstained^29, 35^. Transformants of the mutated *pilBTC* plasmid yielded five red colonies but no transformant of the other two plasmids appeared EPS^+^. The five putative Δ*pilA* suppressor strains were confirmed to be EPS^+^ by an EPS binding assay using the fluorescent dye Calcofluor White. Genetic mapping by transformation using genomic DNA^39, 41^ determined that two out of the five isolates likely had suppressor mutations in the mutagenized *pilBTC* genes as they are linked to the kanamycin resistant (Kan^R^) marker carried by the integrative plasmid. The other three, which must have occurred elsewhere^29, 36, 39, 41, 42^, were not pursued further in this study.

### A Walker B box mutation in PilB suppresses the EPS^−^ phenotype of Δ*pilA*

The mutations in the *pilBTC* gene cluster from the two EPS^+^ strains were identified by cloning and DNA sequencing as described in Methods. The same G to A transition mutation in *pilB* was found in both suppressor strains. This G to A mutation, referred to as pilB* hereafter, occurred at the third position in codon 388 of *pilB*, resulting in a methionine (M) to isoleucine (I) substitution (M388I) (Fig. S3). PilB is an ATPase with the conserved Walker A (WA) and Walker B (WB) boxes^21, 43^ and the M388I substitution resides in its Walker B box (Fig. S3A). Since no other mutation in *pilBTC* was found in the two suppressor strains, they probably originated from the same mutated plasmids.

**Figure 1:**
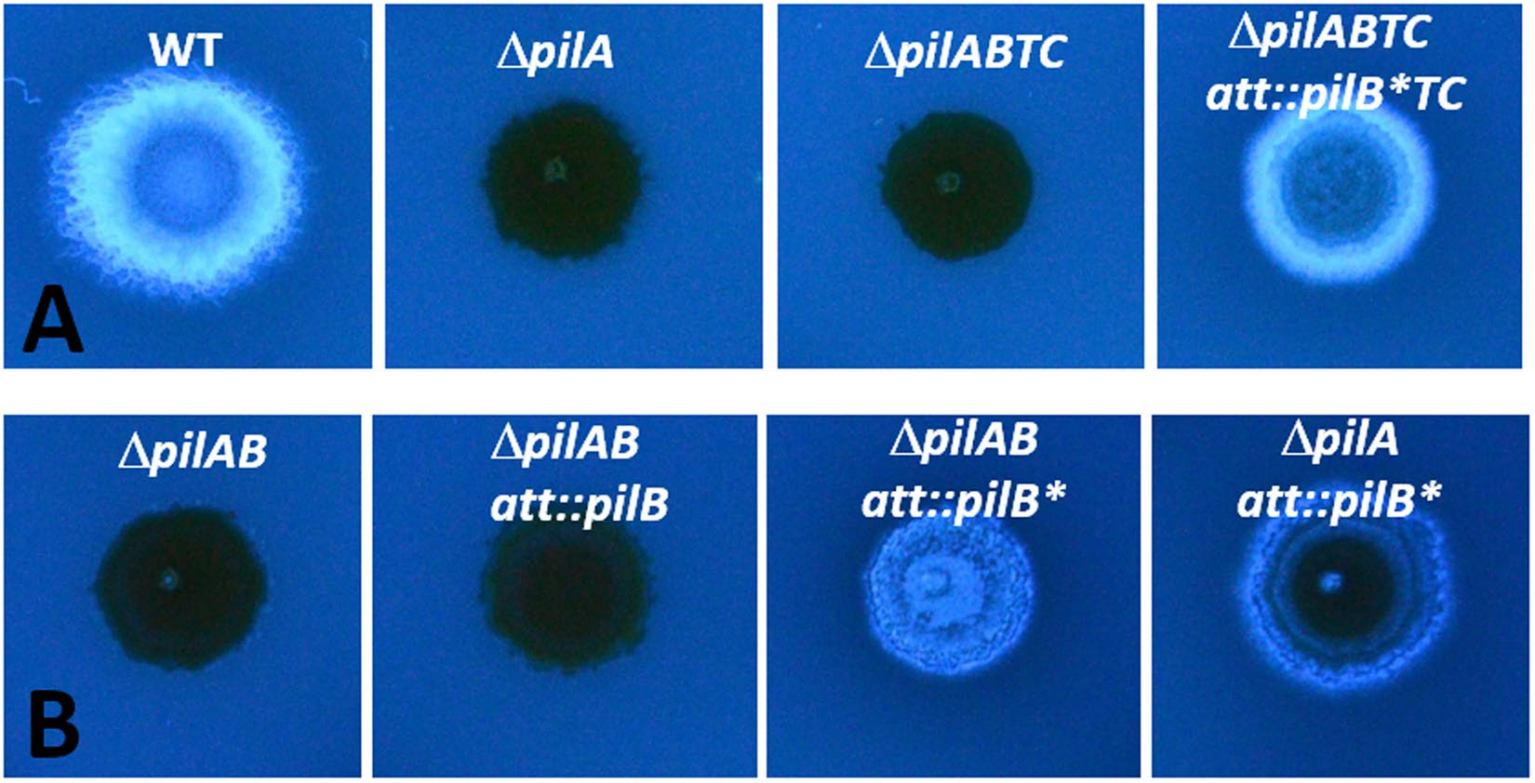
Suppression of Δ*pilA* by pilB*. EPS production was examined on plates with the fluorescent dye Calcofluor white. Panel A: Isolation of pilB* as a suppressor. Stains: DK1622 (WT), YZ690 (Δ*pilA*), YZ1638 (Δ*pilABTC*) and YZ1652 (Δ*pilABTC att*::pilB*TC). Panel B: Confirmation of suppression and dominance of pilB*. Strains: YZ1682 (Δ*pilAB*), YZ1849 (Δ*pilAB att*::*pilB*), YZ1850 (Δ*pilAB att*::pilB*) and YZ1521 (Δ*pilA att*::pilB*). All images in the same panel were from the same plate and the same photograph.

To verify that the pilB* mutation was solely responsible for suppression of Δ*pilA* in the suppressor strains, the *pilB* M388I mutation was reconstructed by targeted mutagenesis in the plasmid containing the WT *pilBTC* gene cluster. When this plasmid with the M388I mutation was transformed into the *pilABTC* quadruple deletion (Δ*pilABTC)* mutant, the resulting strain had its EPS production restored (Fig. 1A). In addition, the M388I mutation was constructed on a plasmid containing *pilB* only. When transformed into a Δ*pilA* Δ*pilB* double mutant, this plasmid restored EPS production to this double deletion strain (Fig. 1B). These results indicate that the pilB* single mutation alone is sufficient to suppress the EPS defect resulting from Δ*pilA*.

### PilB functions upstream of the Dif pathway in EPS regulation

**Figure 2:**
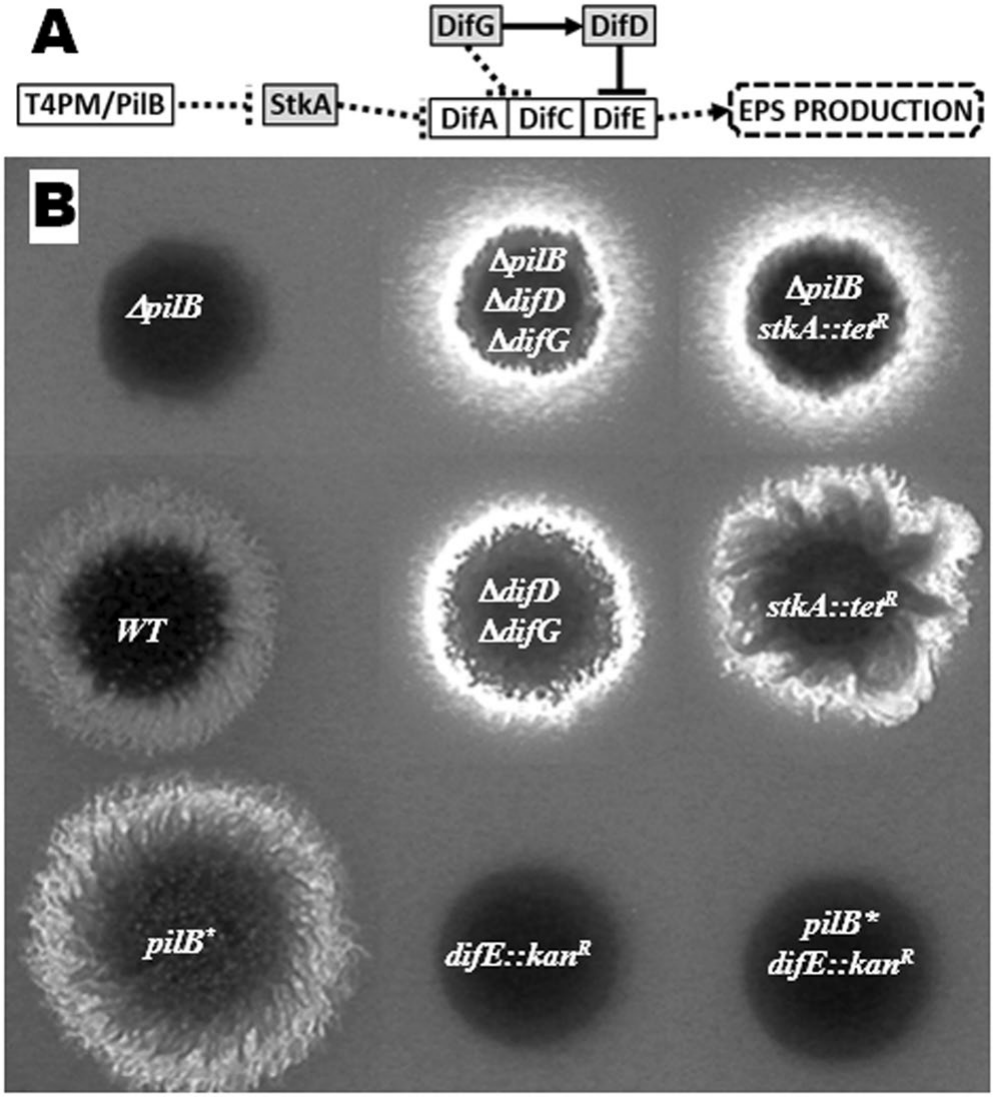
Genetic epistasis between *pilB* and other EPS regulators. Panel A. Model of EPS regulation by T4PM and the Dif pathway in *M. xanthus* ^24, 29–31^. Solid and dash lines represent direct and indirect interactions, respectively. Arrow heads and bars indicate stimulatory and inhibitory effects, respectively. Positive regulators of EPS production are in open boxes and negative one in shaded boxes. The T4PM including PilB functions upstream of StkA and the DifA, DifC and DifE membrane signaling complex. Panel B. EPS production was examined on plates with Calcofluor white. Strains: DK1622 (wild type), DK10416 (Δ*pilB*), YZ1888 (Δ*pilB* Δ*difD* Δ*difG*), YZ1879 (Δ*pilB stkA::tet* ^*R*^), YZ641 (Δ*difD* Δ*difG*), LS2102 (*stkA::tet* ^*R*^), YZ1875 (pilB*), SW501 (*difE::kan* ^*R*^) and YZ1887 (pilB* difE::kan ^*R*^). All images in the same panel were from the same plate and the same photograph.

We examined the relationship between PilB and other known EPS regulators (Fig. 2A) by genetic epistasis (Fig. 2B). *stkA* encodes a DnaK homologue that negatively regulates EPS production^34–36^. A Δ*pilB* Δ*stkA* double mutant was constructed and it was found to be EPS^+^. Likewise, a Δ*difD* Δ*difG* double mutation restored EPS production to a Δ*pilB* mutant as it did to Δ*pilA* ^31^. In contrast, a *difE* ^−^ pilB* double mutant was found to be EPS^−^ similar as the single *difE* ^−^ mutant. Since the suppression of Δ*pilA* by pilB* showed that PilB functions downstream of the T4P filament in EPS regulation, these observations support a model wherein PilB acts as an EPS regulator downstream of the T4PM but upstream of StkA and the Dif pathway^44^.

### PilB* is gain-of-function in EPS regulation albeit WT in S motility

A strain with either the Δ*pilB* mutation or the WT *pilB* (*pilB* ^*WT*^) in a Δ*pilA* background is EPS^−^ (Fig. 1), indicating that neither the *pilB* ^*WT*^ allele nor the null or loss-of-function (LOF) Δ*pilB* mutation can support EPS production in a Δ*pilA* background. The pilB* mutation is therefore likely gain-of-function (GOF) in EPS regulation instead of WT or LOF. If so, it should be dominant over *pilB* ^*WT*^ with regard to the EPS phenotype. To test this, a *pilB* ^*WT*^/pilB* merodiploid was constructed in Δ*pilA* background by transforming the pilB*-containing plasmid into a Δ*pilA* mutant. As shown in Fig. 1B, the resulting strain with these two *pilB* alleles was EPS^+^, indicating pilB* is dominant over *pilB* ^*WT*^ and confirming that pilB* is indeed a GOF mutation with regard to EPS regulation.

**Figure 3:**
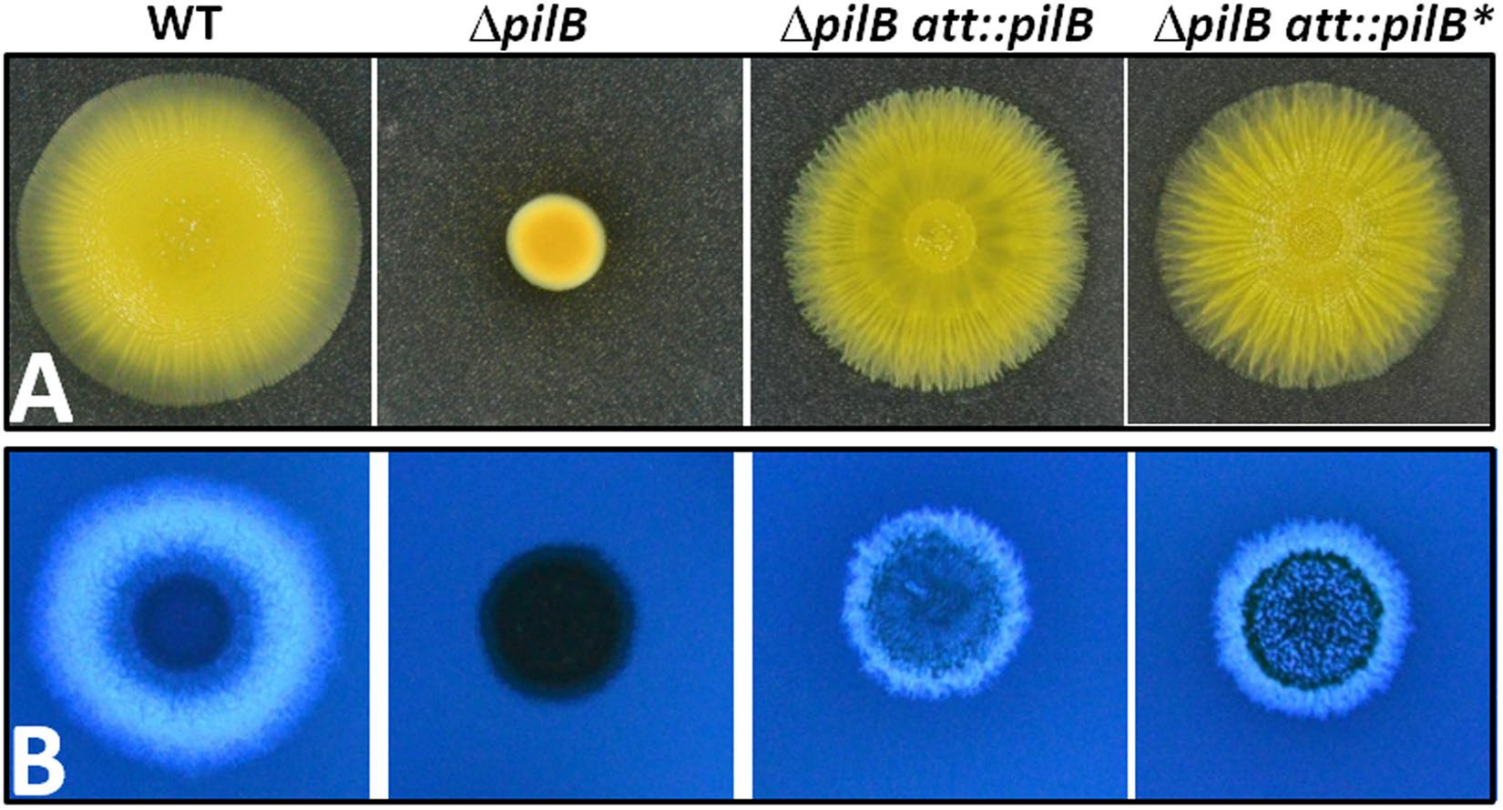
*pil* *B* * complements Δ*pilB* in motility and EPS production. Panela A. S-motility was examined on 0.4% agar plate. Panel B. EPS was analyzed on plates with Calcofluor white. Strains: DK1622 (WT), DK10416 (Δ*pilB*), YZ1674 (Δ*pilB att::pilB*), YZ1522 (ΔpilB att::pilB*).

Since PilB is the T4P assembly motor ATPase, the effect of the pilB* mutation on *M. xanthus* S motility in an otherwise WT background was examined as well. The pilB* and *pilB* ^*WT*^ alleles were introduced into a Δ*pilB* single mutant. The resulting strains were examined for S motility (Fig. 3A) and EPS production (Fig. 3B). As indicated by the clear binding of Calcofluor White in EPS assays, both the p*ilB* ^*WT*^ and pilB* restored EPS production to the *pilB* mutant as expected. Interestingly, pilB* also complemented Δ*pilB* in S motility as analyzed on a soft agar plate. These results demonstrate that while pilB* is a GOF mutation in EPS regulation, it is WT with regard to T4P assembly. PilB* therefore likely retains sufficient ATPase activity to support T4P assembly and S motility *in vivo* despite the significant alteration in its function in EPS regulation. These observations suggest that the ability of PilB to signal EPS production and its role as the T4P assembly ATPase may be genetically separable and functionally distinct.

### An ATPase-deficient PilB variant supports EPS production but not S motility

**Figure 4:**
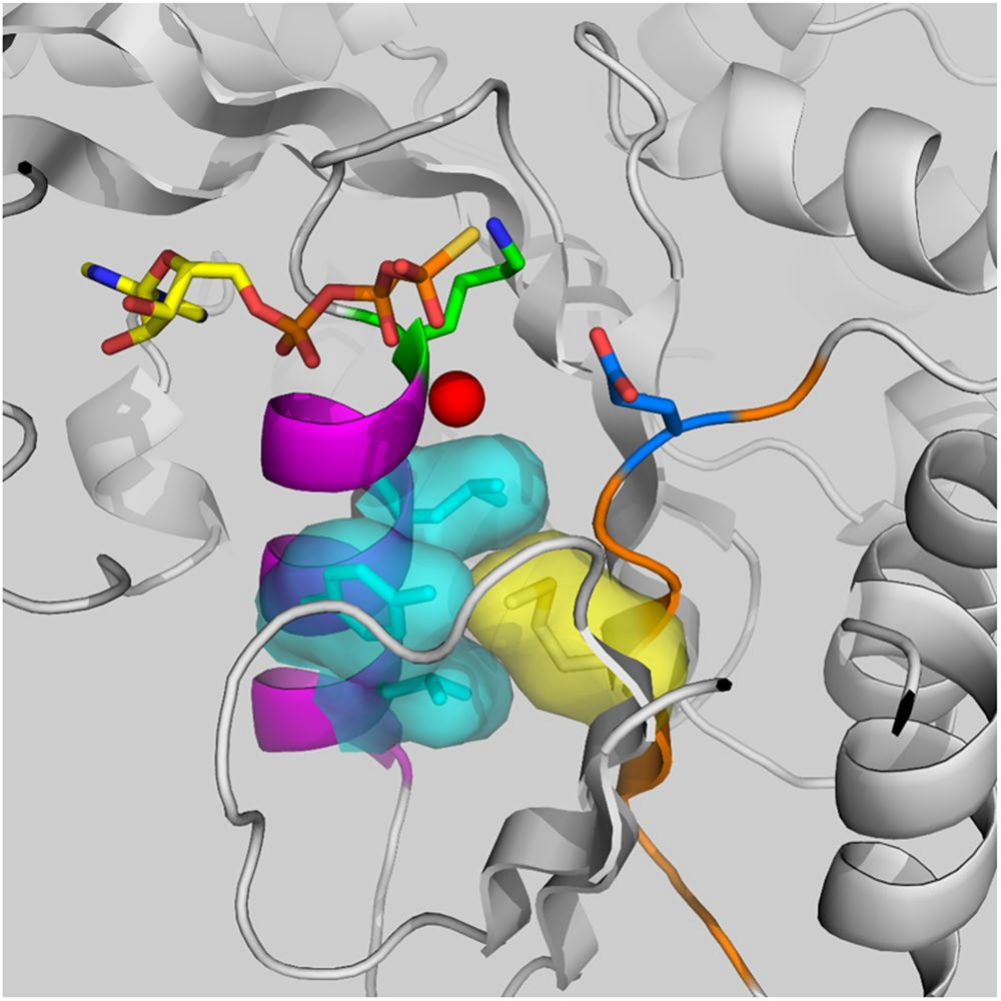
Close-up view of the interactions between Walker A and Walker B. Shown is the MxPilB structure modeled based on TtPilB. The α helix containing WA is colored magenta and the β sheet containing WB is colored orange. The side chain of M388 is shown in yellow and those from the helix residues in close contact with M388 are shown as aqua surfaces. Also shown are the side chains of K327 (green) in WA and E391 (blue) in WB. Because ATP-γ-S and a magnesium ion were present in the TtPilB template structure, we were able to model the ligand (in sticks) and the catalytic magnesium ion (red sphere) as well.

The WA and WB boxes of PilB, while separated by 56 amino acids in the primary sequence (Fig. S3A), are packed against each other in the tertiary structure of the protein^43, 45^. In the model of *M. xanthus* PilB (MxPilB) (Fig. 4), based on the crystal structure of *Thermus thermophilus* PilB/PilF (TtPilB)^43^, the WA box forms the C-terminal part of an α helix and a loop that connects the helix to a β strand. This strand is part of an antiparallel β sheet that lines one side of this α helix. M388 in the WB box is positioned in a strand in the center of this β sheet. The side chain of this methionine packs tightly against the α helix encompassing WA. Substituting this methionine with a bulkier isoleucine in MxPilB or TtPilB is expected to create clashes that would need to be resolved by altering the relative position between WA and WB. Despite its location in the WB box, the M388I substitution therefore may disturb the structure, orientation or conformation of both WA and WB.

**Figure 5:**
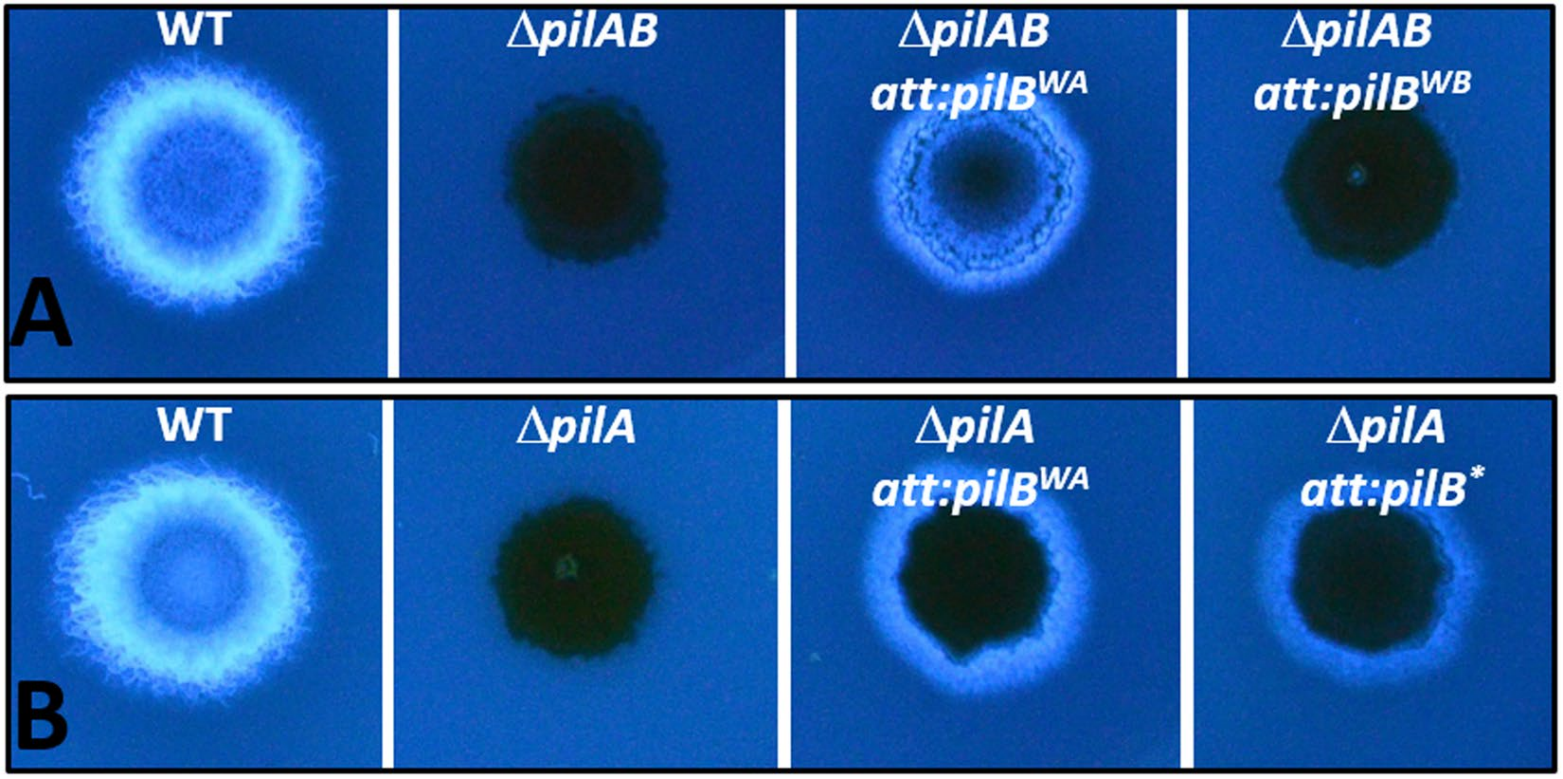
Genetics of *pilB* ^*WA*^ mutation. EPS production was examined on plates with Calcofluor white. Panel A. *pilB* ^*WA*^ suppresses Δ*pilA*. Strains. DK1622 (WT). YZ1682 (Δ*pilA* Δ*pilB*), YZ1504 (Δ*pilA* Δ*pilB att*::*pilB* ^*WA*^), YZ1505 (Δ*pilA* Δ*pilB att*::*pilB* ^*WB*^). Panel B. *pilB* ^*WA*^ is dominant over *pilB* ^*WT*^. Strains: DK1622 (WT), YZ690 (Δ*pilA*), and YZ1513 (Δ*pilA att::pilB* ^*WA*^). YZ1521 (ΔpilA att::pilB*) was included here for comparison. All images in the same panel were from the same plate and the same photograph.

It is known that residues in both WA and WB are crucial for the activity of many ATPases because they interact with the bound ATP molecule^43, 46–48^. Although the M388I mutation apparently does not eliminate the ATPase activity of MxPilB, it may still affect its ATP binding and/or ATPase activity. To test whether the ATPase activity of PilB has an effect on EPS regulation, we constructed mutations known to have more drastic effect on the ATPase activity of PilB. The strictly conserved residues K327 in WA and E391 in WB (Fig. 4) are known to be required for the ATPase activity of MxPilB *in vitro* and its ability to support T4P assembly and S motility *in vivo* ^21^. We constructed K327A (*pilB* ^*WA*^) and E391A (*pilB* ^*WB*^) substitution mutations, respectively, and confirmed that neither mutation supported *M. xanthus* S motility. As shown in Fig. 5A, *pilB* ^*WA*^ but not *pilB* ^*WB*^ restored EPS production to a Δ*pilA* mutant. *pilB* ^*WA*^ is in fact a more robust suppressor than pilB* because its presence in the Δ*pilAB* background results in a higher EPS level when compared to pilB* (Fig. S4). In addition, a *pilB* ^*WA*^ */pilB* ^*WT*^ merodiploid strain in a Δ*pilA* background is EPS^+^ (Fig. 5B), and the dominance of *pilB* ^*WA*^ indicates that it is a GOF mutation in EPS regulation as pilB*. Because PilB^WA^ fails to support S motility *in vivo* and showed no ATPase activity *in vitro* ^21^, the results here (Fig. 5) demonstrate that the role of PilB in EPS production is distinct from its function as the T4P assembly ATPase. That is, in the absence of *pilA*, the catalytically inactive PilB^WA^ not only supports EPS production, but it is even more potent in doing so than its enzymatically active counterparts PilB^WT^ and PilB* (Figs 1, 5 and S4). The stronger EPS phenotype of the *pilB* ^*WA*^ mutant further strengthens the conclusion that the activation of EPS production by PilB does not require or can bypass its function in T4P assembly. These observations support PilB as a signaling or regulatory protein with a more direct role in EPS regulation and that the PilB^WA^ variant may assume a more active signaling conformation without the pilus filament.

### The stability of the PilB* and PilB^WA^ is diminished *in vivo*

**Figure 6:**
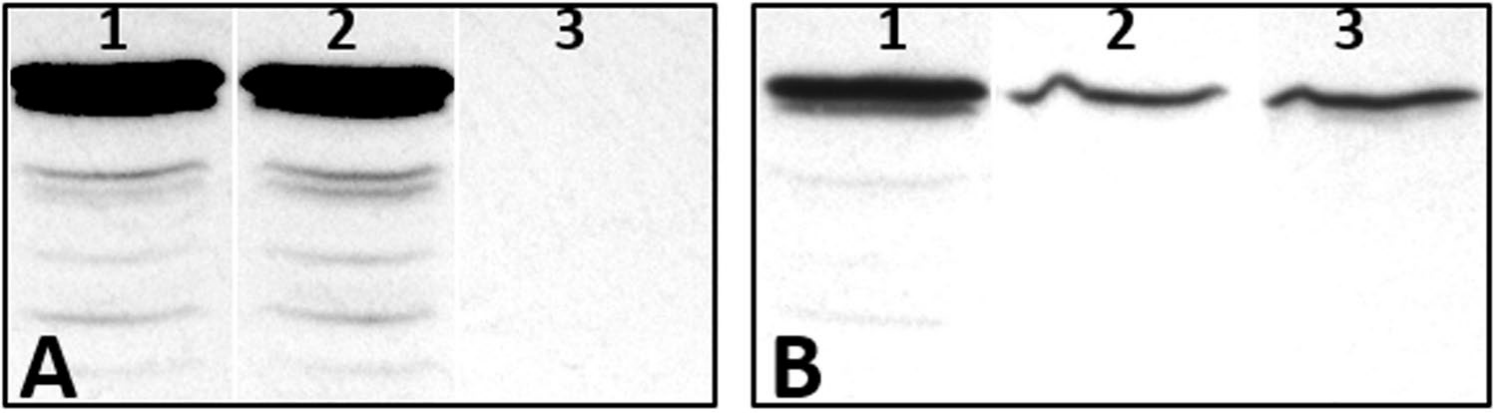
Level of PilB protein examined by immunoblotting. Whole cell lysates from 5 × 10^7^ cells were loaded on each lane and probed with polyclonal anti-PilB antibodies^21^ followed by sencodary antibodies conjugated to horse radish peroxidase. Panel A. Deletion of *pilA* does not affect PilB protein level. Lane 1, DK1622 (WT); Lane 2, DK10407 (Δ*pilA*); Lane 3, YZ1682 (Δ*pilA* Δ*pilB*). Panel B. Levels of PilB* and PilB^WA^ are reduced. Lane 1, YZ1849 (Δ*pilA* Δ*pilB att*::*pilB*); Lane 2, YZ1850 (Δ*pilA* Δ*pilB att*::pilB*); Lane 3, YZ1504 (Δ*pilA* Δ*pilB att*::*pilB* ^WA^). The samples in each panel were from the same immunoblot with the same exposure although not on contiguous lanes. The full immunoblots are provided as Fig. S5.

We explored the possibility that the EPS^+^ phenotype of pilB* and *pilB* ^*WA*^ mutations may have been due to altered the protein level of PilB by performing immunoblotting using anti-PilB antibodies^21^. The levels of PilB in the WT and the Δ*pilA* backgrounds were virtually indistinguishable (Fig. 6A), suggesting that the Δ*pilA* mutation itself does not alter PilB expression or stability. However, the levels of both PilB* and PilB^WA^ were lower than PilB^WT^ in an isogenic Δ*pilA* background (Fig. 6B). Since the experiments were conducted with strains where all *pilB* variants were expressed from the same promoter^49^, it is unlikely that transcription or translation are affected by these mutations. Instead, it is more likely that the stability of the protein *in vivo* was changed by these mutations. Regardless, since a *pilB* null mutation does not suppress the EPS^−^ phenotype of Δ*pilA*, only an increase in PilB protein level could explain the suppression of Δ*pilA* by pilB* and *pilB* ^*WA*^, but not a decrease. We propose that PilB* and PilB^WA^ mutations changed the structure of the protein to favor its active signaling conformation and that this conformational change may have reduced the stability of PilB *in vivo* coincidentally.

### PilB^WA^ is altered in its structure and response to ATP

To gain insights into the mechanisms of PilB in EPS regulation, we attempted to purify and examine the properties of PilB^WT^, PilB^WA^ and PilB^WB^ proteins *in vitro*. These three PilB variants were chosen because they reflect the three distinct phenotypes with regard to T4P assembly and EPS production *in vivo*. PilB* was not included in the *in vitro* studies because it resulted in an intermediate EPS phenotype. Purified MxPilB exhibits measurable but very low ATPase activity^21^ and it tends to form protein aggregates (unpublished). We henceforth expressed and purified the *T. thermophilus* PilB^WT^, PilB^WA^ and PilB^WB^ equivalents (Fig. S3A)^50^ for *in vitro* studies.

**Figure 7:**
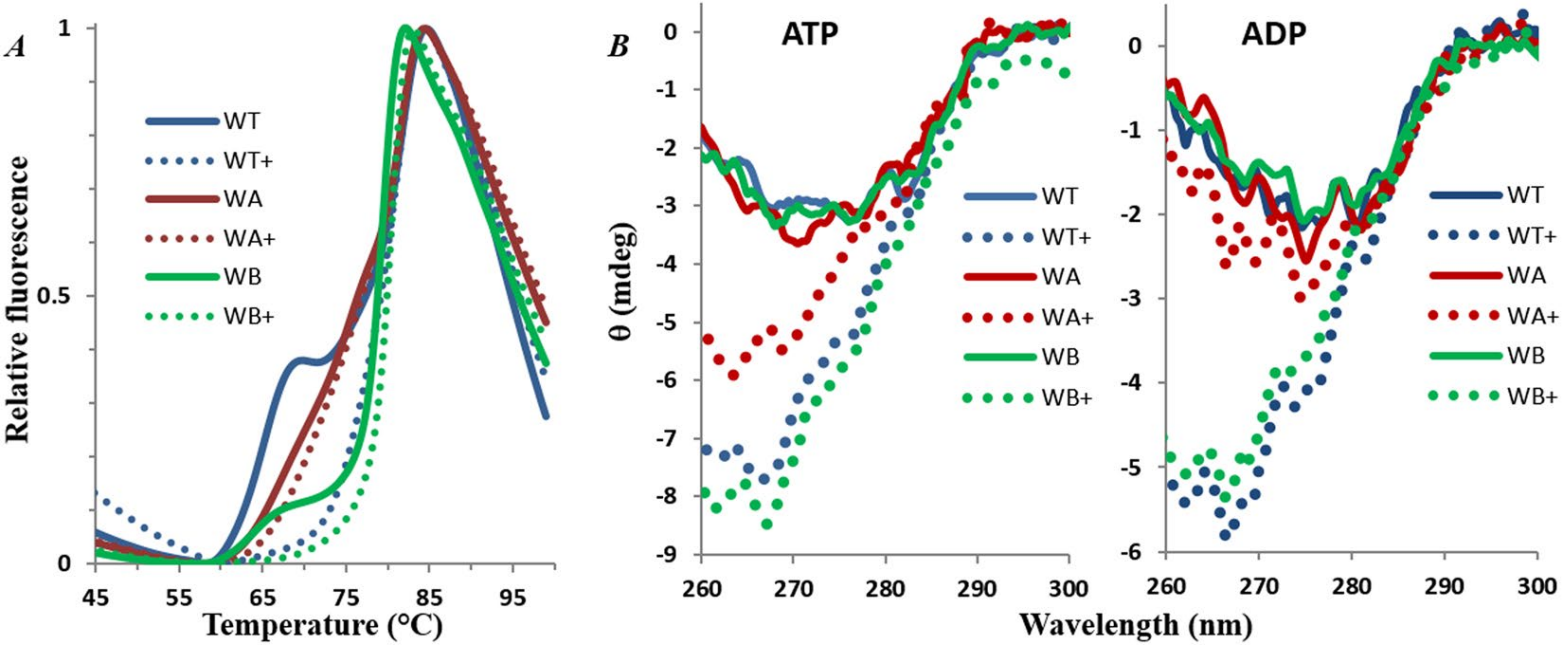
*T. thermophilus* PilB^WA^ is structurally distinct from PilB^WT^ and PilB^WB^. PilB proteins were analyzed by DSF (Panel A) and near UV CD (Panel B) (See Methods). The fluorescence intensity is normalized between 0 and 1 in Panel A and the ellipticity (θ) is expressed in milli degrees (mdeg) in Panel B. For both panels, WT stands for PilB^WT^, WA for PilB^WA^ and WB for PilB^WB^. The plus sign (+) indicates supplementation of 0.4 mM AMP-PNP in Panel A and 0.1 mM ATP (left) or ADP (right) in Panel B.

Differential scanning fluorimetry (DSF), which measures the thermal denauration of a protein over a temperature gradient^51, 52^, was used to first examine the structural differences among PilB^WT^, PilB^WA^ and PilB^WB^. As shown in Fig. 7A, PilB^WT^ and PilB^WB^ behaved relatively similar in this assay: both show biphasic unfolding profiles with two melting temperatures or transition midpoints (*T*_*m*_) around 64–65 °C and 79–81 °C, respectively. In contrast, PilB^WA^ unfolded over a broader temperature range with an apparent *T*_*m*_ around 76 °C. The obvious differences in thermal stability indicate that the structure of PilB^WA^, but not that of PilB^WB^, is significantly altered in comparison with PilB^WT^. We also examined the effect of AMP-PNP, a non-hydrolyzable ATP analogue^53, 54^, on PilB thermal stability (Fig. 7A). This nucleotide stabilized both PilB^WT^ and PilB^WB^, resulting in the disappearance of the first unfolding transition. In contrast, the stability of PilB^WA^ remained virtually unchanged by the addition of AMP-PNP. These results demonstrate that PilB^WA^ no longer responds to its ligands and that its structure is distinct from those of PilB^WT^ and PilB^WB^.

The circular dichroism (CD) spectra of these protein variants were collect to further explore their structural differences. In the far ultraviolet (UV) range (200–240 nm), all three proteins showed similar spectra with or without ATP or ADP (Fig. S7). As the signals in this range are sensitive to changes in protein secondary structure^55^, the results indicate that the secondary structure content is unaffected by the mutations in PilB^WA^ and PilB^WB^ or by ligand binding. In the near UV range (260–320 nm), changes in the CD spectra are related to protein conformation^55^. The signals in the 260–285 nm range are contributed primarily by phenylalanine and tyrosine residues^55^, which the TtPilB contains 14 each (Fig. S3A). There is little signal beyond 285 nm because the protein lacks tryptophan (Figs 7B and S3A). All three protein variants, PilB^WT^, PilB^WA^ and PilB^WB^, showed similar CD spectrum without ligand. While the addition of ATP shifted the spectra of all three proteins downward in the 260–280 nm range (Fig. 7B, left panel), the shift for PilB^WA^ is less pronounced compared to those seen for the other two. The addition of ADP also shifted the spectra of PilB^WT^ and PilB^WB^, but not that of PilB^WA^ (Fig. 7B, right panel). These observations further underscored that PilB^WA^ is diminished in its response to nucleotides when compared to PilB^WT^ and PilB^WB^. In particular, PilB^WA^ more closely resembles the ligand-free rather than the ligand-bound form of PilB in the presence of a nucleotide.

**Figure 8:**
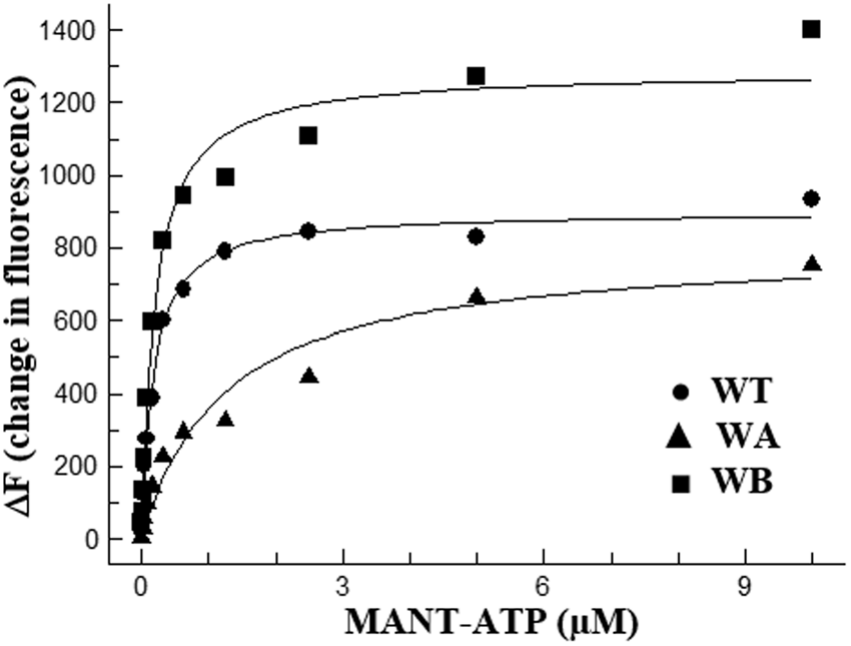
PilB^WA^ shows reduced binding affinity for nucleotide. PilB protein at 0.2 μM was titrated with increasing concentrations of the fluorescent ATP analog MANT-ATP and data were collected and analyzed (See Methods). Dots (●) represent data points for (WT) PilB^WT^ (WT), triangles (▲) for PilB^WA^ (WA) and squares (■) for PilB^WB^ (WB). The best fitting curve is shown for each data set.

The binding affinity of PilB for ATP was examined using the ATP analogue MANT-ATP^56^. The fluorescence of this nucleotide is enhanced by a more hydrophobic environment frequently associated with its binding to a protein. Figure 8 shows the difference in fluorescence (ΔF) in the presence and absence of PilB variants over varying concentrations of MANT-ATP. A binding isotherm was fit to the data to estimate the binding affinity of the three PilB protein variants to this ATP analogue. The dissociation constants (K_d_) for PilB^WT^ and PilB^WB^ were similar with values of 0.17 μM and 0.19 μM, respectively. The K_d_ for PilB^WA^ is 1.23 μM and this increase in K_d_ represents a significant reduction in its nucleotide binding affinity. These results suggest that the diminished response of PilB^WA^ to nucleotides is attributed to its reduced affinity for its ligand. The *in vitro* studies (Figs 7 and 8) suggest that the nucleotide-free conformation of PilB may actively signal EPS production in *M. xanthus*.

## Discussion

This study identified the T4P assembly ATPase PilB as a regulator of EPS downstream of the T4P filament and upstream of the Dif signaling proteins in *M. xanthus*. A genetic screening was devised to first isolate *pil* mutations that could restore EPS production to a Δ*pil*A strain, leading to the discovery of pilB* (Fig. 1). Further genetic analysis indicated that PilB functions downstream of T4PM but upstream of the StkA and Dif proteins in EPS regulation (Fig. 2)^44^. Targeted mutagenesis of conserved residues revealed that the involvement of PilB in EPS regulation can be separated from and is independent of its function as the T4P assembly ATPase (Fig. 5). That is, the PilB^WA^ mutant variant, which does not hydrolyze ATP or support S motility^21^, may be locked in a conformation that actively signals EPS production in a Δ*pilA* or a WT background (Fig. 5). Both pilB* and *pilB* ^*WA*^ are dominant over *pilB* ^*WT*^ (Figs 1 and 5), indicating that they are GOF mutations. *In vitro* studies using TtPilB indicated that the PilB^WA^ variant showed diminished conformational response to the addition of nucleotides because of reduced binding affinity (Figs 7 and 8). These results lead to a model wherein the *M. xanthus* PilB ATPase functions as a signaling protein in EPS regulation and its nucleotide-free state may correlate with its actively signaling conformation.

As far as we are aware, XB24^57^ and the Na/K ATPase (NKA)^58, 59^ are the two other cases where ATPases have been proposed as signaling proteins. XB24 is a small cytoplasmic ATPase in rice with a role in immunity and defense against bacterial pathogens. It physically associates with and modulates the activity of the kinase XA21, which is a receptor for recognition of pathogen-associated molecular patterns (PAMPs). The ATPase activity of XB24 is required for its function *in vivo* and it is proposed that the signaling activity of XA21 is regulated by XB24. NKA is ubiquitous in the plasma membrane of all animal cells. It establishes and maintains the ion homeostasis essential for cell viability. All NKAs contain a binding site specific for steroid inhibitors such as ouabain and digoxin. There is evidence that NKAs can associate with and affect the activity of signaling proteins such as the Src kinase^60, 61^. It has been proposed that NKAs are receptors and signal transducers to regulate downstream targets in a signal transduction pathway.

Drawing analogy between G-proteins^62, 63^ and signaling ATPases^57, 58^, we hypothesized initially that it was the PilB in the ATP bound form that actively signaled EPS production. This was tested by mutating E391 in WB, a key catalytic residue for ATP hydrolysis^47, 48^ (Fig. 4). Since PilB^WB^ has the same binding affinity for ATP as the PilB^WT^ (Fig. 8) but is inactive as an ATPase^21^, it likely exists in its ATP-bound form in the cell. The *pilB* ^*WB*^ mutation, however, failed to suppress Δ*pilA* (Fig. 5), arguing against the ATP-bound PilB as the actively signaling conformation. On the other hand, the highly conserved K327 in WA is known to be critical for ATP binding^47, 48^ (Fig. 4). When mutated to an alanine, the resulting *pilB* ^*WA*^ turned out to be a robust suppressor of Δ*pilA* in EPS regulation (Figs 5 and S4). Binding assays indicated that PilB^WA^ binds to ATP with much reduced affinity as expected (Fig. 8). Biophysical studies also suggested that the structure of PilB^WA^ resembles the apo-form of PilB^WT^ with or without its nucleotide ligands (Fig. 7B). We therefore propose that it is the apo form of PilB that actively signals EPS production in *M. xanthus*. It remains to be seen how prevalent signaling ATPases are in different biological systems and whether they share a common signaling conformation with *M. xanthus* PilB.

The regulation of EPS is the key aspect of bacterial biofilm formation^64–66^. The paradigm that emerged from the studies of biofilm in flagellated motile bacteria is the mutual exclusivity or inverse regulation of the motile state vs the biofilm state^64–66^. That is, the regulation is such that the motility of cells in a biofilm is inhibited whereas motile cells either exit from or exist mostly outside of biofilms in bacteria with flagellated motility. The small signaling molecule c-di-GMP is well known as the “master” regulator of the transition between the motile and the biofilm states. The overarching conclusion from studies of this signaling molecule is that it simultaneously enhances biofilm formation and inhibits flagellated motility. The known effectors or targets of c-di-GMP in biofilm regulation are diverse, ranging from biosynthetic enzymes and riboswitches to transcriptional activators and posttranslational regulators^65^. Nevertheless, EPS, the major constituent of bacterial biofilms, is the ultimate target of this regulation in most cases. For the regulation of flagellated motility, c-di-GMP generally targets the expression or activity of flagellar proteins. In *V. cholerae*, for example, such regulations are achieved by the c-di-GMP receptors and transcriptional regulators VpsR and FlrA/FliQ among others^67^. VpsR, which directly activates the expression of EPS or VPS (*V* *ibrio* polysaccharides) genes, binds c-di-GMP with high affinity. FlrA/FliQ is the master regulator of flagellar genes and the binding of c-di-GMP impairs its ability to activate the transcription of the flagellar operon.

More recently, a new c-di-GMP binding motif was discovered by the studies of *V. cholerae* MshE, the PilB equivalent in the Msh pilus system^68–70^. Sequence alignment with MshE shows convincingly that PilB from *M. xanthus* and many other T4P systems contain this c-di-GMP binding motif at their N-termini (Fig. S3B)^68^. This motif by itself and a few proteins with it have been verified or demonstrated to bind c-di-GMP with high affinity^68^. These include a PilB from *Clostridium perfringens* ^71^. It is therefore reasonable to assume that *M. xanthus* PilB is a functional c-di-GMP effector. With this in mind, the finding of *M. xanthus* PilB as an EPS regulator here suggests that c-di-GMP manages biofilm formation and T4P-mediated S motility through PilB as a direct target in *M. xanthus*. Drawing analogies with flagellated bacteria, we envision a similar working model for the regulation of EPS production and S motility by c-di-GMP in *M. xanthus*. We propose that c-di-GMP promotes EPS production and biofilm formation at high concentrations whereas the T4P-dependent S motility is favored at low or basal levels of c-di-GMP. In this model, when c-di-GMP is present at low or basal levels, PilB is active as the T4P assembly ATPase while EPS is produced only at a basal level to allow *M. xanthus* S motility to function^26, 72, 73^. When the cellular concentration of c-di-GMP is high, it binds to PilB to signal EPS production and to inhibit its activity as the T4P assembly motor simultaneously. We suggest that the binding c-di-GMP results in conformational changes in PilB mimicked or represented by the PilB^WA^ mutant protein, and such conformational changes lead to the inhibition of ATPase activity and stimulation of EPS signaling.

## Methods

### Growth Conditions

*Myxococcus xanthus* strains used in this study are listed in Table 1. They were grown and maintained at 32 °C on Casitone-yeast extract (CYE) agar plates or in CYE liquid medium^74^. XL1-Blue (Stratagene) and Rosetta (Novagen), the *Escherichia coli* strains used for plasmid construction and protein expression, were grown and maintained at 37 °C on Luria-Bertani (LB) agar plates or in LB liquid medium^75^. Unless noted otherwise, plates contained 1.5% agar. Kanamycin and ampicillin at 100 μg/ml and oxytetracycline at 15 μg/ml were added to media for selection when appropriate.

**Table 1:** *M. xanthus* strains.

Strains	Relevant genotype or description†	Source or reference
DK1622	Wild type	20
DK10416	Δ*pilB*	18
LS1102	*stkA::tet* ^*R*^ *)*	35
SW501	*difE::kan* ^*R*^	27
YZ641	Δ*difD* Δ*difG*	31
YZ690	Δ*pilA*	This study
YZ1504	Δ*pilB* Δ*pilA att::pilB* ^*WA*^	This study
YZ1505	Δ*pilB* Δ*pilA att::pilB* ^*WB*^	This study
YZ1512	Δ*pilB att::pilB* ^*WA*^	This study
YZ1513	Δ*pilA att::pilB* ^*WB*^	This study
YZ1520	Δ*pilB att::pilB* ^*AB*^	This study
YZ1521	ΔpilA att::pilB*	This study
YZ1522	ΔpilB att::pilB*	This study
YZ1548	Δ*pilB att::pilB* ^*WB*^	This study
YZ1636	Δ*pilBTC*	This study
YZ1637	Δ*pilMNOPQ*	This study
YZ1638	Δ*pilBTC* Δ*pilA*	This study
YZ1639	Δ*pilAGHI*	This study
YZ1640	Δ*pilMNOPQ* Δ*pilA*	This study
YZ1644	Δ*pilBTC att::pilBTC*	This study
YZ1645	Δ*pilMNOPQ att::pilMNOPQ*	This study
YZ1638	Δ*pilBTC* Δ*pilA*	This study
YZ1652	Δ*pilBTC* ΔpilA att::pilB*TC	This study
YZ1674	Δ*pilB att::pilB*	This study
YZ1682	Δ*pilB* Δ*pilA*	This study
YZ1849	Δ*pilB* Δ*pilA att::pilB*	This study
YZ1850	Δ*pilB* ΔpilA att::pilB*	This study
YZ1865	Δ*pilGHI*	This study
YZ1870	Δ*pilGHI att::pilGHI*	This study
YZ1875	pilB*	This study
YZ1879	Δ*pilB stkA::tet* ^*R*^	This study
YZ1887	pilB* difE::kan ^*R*^	This study
YZ1888	Δ*pilB* Δ*difD* Δ*difG*	This study

^**†**^ *pilB* ^*WA*^ refers to the K327A mutation in Walker A, *pilB* ^*WB*^ to the E391A in Walker B, and pilB* to the M388I original suppressor mutation (also see Fig. S3).

### Plasmid used for strain construction

Three sets of plasmids were generated to construct *M. xanthus* strains. The first set were constructs to delete single or multiple *pil* genes; these include pWB525 (Δ*pilA*), pWB581 (Δ*pilB*), pWB555 (Δ*pilBTC*), pWB556 (Δ*pilAGHI*), pWB605 (Δ*pilGHI*) and pWB557 (Δ*pilMNOPQ*). The second were for the expression of *pil* gene clusters and *pilB* alleles in *M. xanthus*; these constructs, which integrate at Mx8 *att* site^76^, include pWB559 (*pilGHI*), pWB565 (*pilMNOPQ*) and pWB566 (*pilBTC*) as well as pWB571 (*pilB*), pWB572 (pilB*), pGD5 (*pilBWA*) and pGD6 (*pilBWB*). The third includes the plasmid pWB606, which was used for the replacement of wild-type *pilB* with pilB*. The first and third sets were derivatives of pBJ113^77^. The second set used pWB425 as the cloning and *M. xanthus* expression vector^39^. pWB425 contains a BspHI/ApoI fragment from pZero-2 (Invitrogen) with the kanamycin resistance (Kan^R^) gene and its promoter. The expression of all *pil* genes cloned into pWB425 is driven by this promoter because the multicloning site is immediately downstream of the Kan^R^ gene in this plasmid.

### Plasmid construction and description

The details for the construction of plasmids used for *M. xanthus* strain construction are described here. Fragments with in-frame deletion alleles of *pilA*, *pilBTC*, *pilGHI*, *pilAGHI* and *pilMNOPQ* were generated by a two-step overlap PCR as described previously^29^. These fragments were cloned into pBJ113^77^ to construct pWB525 (Δ*pilA*), pWB555 (Δ*pilBTC*), pWB556 (Δ*pilAGHI*), pWB605 (Δ*pilGHI*) and pWB557 (Δ*pilMNOPQ*). In these plasmids, the Δ*pilA* allele deleted the codons from 7 to 218 of *pilA*, Δ*pilBTC* from 8 of *pilB* to 409 of *pilC*, Δ*pilAGHI* from 7 of *pilA* to 250 of *pilI*, Δ*pilGHI* from 5 of *pilG* to 250 of *pilI* and Δ*pilMNOPQ* from 7 of *pilM* to 896 of *pilQ*, respectively. The *pilB* deletion allele from DK10416^18^ was PCR amplified and cloned into pBJ113 to produce pWB581. For the allelic exchange of wild-type *pilB* with pilB*, the fragment from pWB572 (see below) was cloned into pBJ113 to create pWB606.

Plasmids that can integrate into *M. xanthus* chromosome at Mx8 phage attachment site (*att*)^78^ for ectopic expression were constructed using pWB425^39^ as the vector. pWB559, pWB565 and pWB566, which contain the respective *pilGHI, pilMNOPQ* and *pilBTC* gene clusters from pDW79^79^ or pSWU257^17^ in pWB425, were also used for mutagenesis in *E. coli*. With reference to the coding regions (Fig. S1), pWB559 (*pilGHI*) contains DNA from 73 bp upstream of *pilG* to 328 bp downstream of *pilI*, pWB565 (*pilMNOPQ*) from 120 bp upstream of *pilM* to 19 bp downstream of *pilQ*, and pWB566 (*pilBTC*) from 22 bp upstream of *pilB* to 5 bp downstream of *pilC*. In addition, pWB571 (*pilB*), which contains from 22 bp upstream to 142 bp downstream of *pilB*, was derived from pWB566. This was achieved by digestion with SacI (site in *pilT*) and BamHI (site on vector), treatment with T4 DNA polymerase and religation; this removed *pilC* and the bulk of *pilT*. pWB572 (pilB*), pGD5 (*pilB* ^*WA*^) and pGD6 (*pilB* ^*WB*^) were constructed using pWB571 as a template by a two*-*step overlap PCR using primers containing the M388I (pilB*), K327A (*pilB* ^*WA*^) and E391A (*pilB* ^*WB*^) mutations.

### Strain Construction

YZ690 through YZ1888 in Table 1 are the *M. xanthus* strains constructed in this study. For the construction of the *pil* deletions and the pilB* chromosomal replacement mutant, the plasmids with these mutant alleles were electroporated into DK1622 and used for allelic exchange as previously described^29, 80, 81^. These strains are YZ690 (Δ*pilA*), YZ1636 (Δ*pilBTC*), YZ1637 (Δ*pilMNOPQ*), YZ1639 (Δ*pilAGHI*), YZ1865 (Δ*pilGHI*) and YZ1875 (pilB*). The deletion strains YZ1638 (Δ*pilBTC* Δ*pilA*), YZ1640 (Δ*pilMNOPQ* Δ*pilA*) and YZ1682 (Δ*pilB* Δ*pilA*) were constructed similarly using YZ690 (Δ*pilA*) as the parent.

The plasmids constructed with pWB425 as the vector were electroporated into the appropriate deletion strains to construct YZ1644 (Δ*pilBTC att::pilBTC*), YZ1645 (Δ*pilMNOPQ att::pilMNOPQ*) and YZ1870 (Δ*pilGHI att::pilGHI*). Other strains similarly constructed are YZ1504 (Δ*pilB* Δ*pilA att::pilB* ^*WA*^), YZ1505 (Δ*pilB* Δ*pilA att::pilB* ^*WB*^), YZ1849 (Δ*pilB* Δ*pilA att::pilB*) and YZ1850 (Δ*pilB* ΔpilA att::pilB*) as well as YZ1512 (Δ*pilB att::pilB* ^*WA*^), YZ1522 (ΔpilB att::pilB*), YZ1548 (Δ*pilB att::pilB* ^*WB*^) and YZ1674 (Δ*pilB att::pilB* ^*WB*^). The *pilB* merodiploid strain YZ1521 (ΔpilA att::pilB*) was constructed by transforming pWB572 into YZ690 (Δ*pilA*).

YZ1879 (Δ*pilB stkA::tet* ^*R*^) and YZ1887 (pilB* difE::kan ^*R*^) were constructed by transferring the *stkA::tet* ^*R*^ allele in LS1102 to DK10416 (Δ*pilB*) and the *difE::kan* ^*R*^ allele in SW501 to YZ1875 (pilB*) by genomic transformation^41^, respectively. pWB581 (Δ*pilB)* was used to replace *pilB* ^*WT*^ with Δ*pilB* in YZ641 (Δ*difD* Δ*difG*) to construct YZ1888 (Δ*pilB* Δ*difD* Δ*difG*).

### Mutagenesis and suppressor identification

pWB559, pWB565 and pWB566 were mutagenized by propagation in NR9458, an *E*. *coli mutD5* mutator strain^40^. Cells were initially grown on plates with 1 × Volgel-Bonner salts minimal media containing 0.4% glucose, 50 μg/ml proline and 5 μg/ml thiamine to minimize the mutation rate^40^. Plates containing approximately 100 colonies for each plasmid to be mutagenized were pooled and inoculated into LB broth to increase the mutation rate. Mutagenized pools of plasmid DNA were prepared from overnight cultures and used to transform the appropriate *pil* deletion strains. Potential EPS producing suppressor mutants were identified as reddish-orange colonies on CYE plates containing Congo red (30 μg/ml)^35^. Genomic DNA from potential suppressor mutants was electroporated into the original parental strain and selected on plates with kanamycin and Congo red to examine the link of the EPS phenotype with the integrated plasmid^39, 41^. Genomic DNA from suppressor mutants was cut with PstI, religation and transformation into *E*. *coli* XL1-Blue to recover the plasmid and *pil* mutations were identified by DNA sequencing.

### Phenotypic analysis

Log phase cells grown in CYE were harvested and resuspended in MOPS buffer (10 mM morpholinopropanesulfonic acid [pH 7.6], 2 mM MgSO_4_) at 5 × 10^9^ cells/ml. 5 µl of this cell suspension was spotted onto CYE plates with 0.4% agar and regular CYE plates with Calcofluor white (50 µg/ml) for S-motility and EPS analysis, respectively. Plates were incubated at 32 °C for 5 days before documentation under white light for S motility and 365 nm UV light for EPS production.

### Protein purification

Plasmids pWB750, pWB751 and pWB752 (see SI Materials and Methods) were constructed and maintained in the *E*. *coli* strain XL1-Blue initially. For protein purification, they were transformed into the *E*. *coli* Rosetta strain containing pREP4 (Qiagen). The appropriate expression strains were grown at 35 °C to an OD_600_ of 0.5–0.6 in 1 liter of LB plus ampicillin (100 μg/ml) and kanamycin (25 μg/ml). Protein expression was induced by addition of IPTG (Isopropyl β-D-1-thiogalactopyranoside) to a final concentration of 0.1 mM, followed by incubation at 30 °C for 4–5 hrs. Please see SI Materials and Methods for more details for the purification.

### Differential Scanning Fluorimetry (DSF)

Proteins were diluted to a final concentration of 8 μM in 1× stock buffer (10 mM HEPES [pH 8], 50 mM KCl, 10 mM MgCl_2_, 0.5 mM EDTA, 1 mM β-ME and 10% glycerol) using a 5× stock buffer. Sypro Orange (Ex. 490 nm, Em. 530 nm) from a 5,000× stock (Invitrogen) was diluted to 50× in deionized water and used at a 5× concentration in DSF assays. For examining the effects of ligand, adenosine 5′-(β,γ-imido)triphosphate (AMP-PNP) (Sigma-Aldrich) was added to a final concentration of 0.4 mM. The experiment was carried out using a Bio-Rad CSX96 Real-Time System (Bio-Rad), starting at 25 °C with temperature increments of 0.5 °C to a final temperature of 99 °C. Samples were held at each temperature for 30 sec prior to measurement of fluorescence. Data were analyzed and Tm’s were obtained using Bio-Rad’s CFX Manager software. The data points starting at 45 °C were presented in this paper.

### Circular Dichroism (CD)

Protein samples were adjusted to a final concentration of about 6 µM for far UV (200–260 nm) by diluting in storage buffer. For near UV (260–320 nm), proteins were concentrating using Amicon stirred cells to 230 µM and 170 µM for the experiment with ATP and ADP, respectively. CD spectra were generated on a Jasco J-815 Spectropolarimeter equipped with the Jasco PFD-425S temperature-control unit in a 1-mm path-length quartz cell at 25 °C. Each spectrum was from three accumulated scans with a 1 second response time at a scan speed of 100 nm per minute. Scans were performed with a bandwidth of 1 nm in far UV and at 0.5 nm increments in near UV. Data was recorded using Spectra Manager software (Jasco). For examining the effects of ligand, ATP and ADP (Sigma-Aldrich) was added at a final concentration of 0.1 mM. Data was plotted after background subtraction of identical scans of controls without the protein. ATP and ADP were used in this experiment because these *T. thermophilus* PilB variants has no ATPase activity at 25 °C (data not shown).

### Nucleotide Binding Assay

Binding of MANT-ATP [2′-/3′-O-(N-methylanthraniloyl) adenosine-5′-triphosphate] (Invitrogen) was monitored by measurement of fluorescent intensity using an Infinite M200 microplate reader (Tecan). The excitation was set to 356 nm and fluorescence emission was recorded at 448 nm. Samples with MANT-ATP at specified concentrations (0.0012 to 10 μM) were incubated with or without the protein at 0.2 μM at room temperature for 2 hours in binding buffer [10 mM Tris (pH 9), 50 mM KOAc, 5 mM MgOAc, and 1% glycerol] prior to fluorescence measurements. The difference in fluorescence (ΔF) with and without the protein at different MANT-ATP concentrations ([MANT-ATP]) was fitted to the binding isotherm ΔF = ΔF_max_ * [MANT-ATP]/(K_d_ + [MANT-ATP]) where ΔF_max_ is the maximum ΔF and K_d_ is the dissociation constant. Curve fitting and determination of K_d_ values were performed using XLfit (ID Business Solutions).

## Electronic supplementary material

Supplemental Information

## References

[1] Kaiser D, Manoil C, Dworkin M (1979). Myxobacteria: cell interactions, genetics, and development. Annual review of microbiology.

[2] Shimkets LJ (1990). Social and developmental biology of the myxobacteria. Microbiol Rev.

[3] Berleman JE, Scott J, Chumley T, Kirby JR (2008). Predataxis behavior in *Myxococcus xanthus*. Proceedings of the National Academy of Sciences of the United States of America.

[4] Zusman DR, Scott AE, Yang Z, Kirby JR (2007). Chemosensory pathways, motility and development in *Myxococcus xanthus*. Nat Rev Microbiol.

[5] Kaiser D (2008). *Myxococcus:* From Single-Cell Polarity to Complex Multicellular Patterns. Annu Rev Genet.

[6] Berleman JE (2016). Exopolysaccharide microchannels direct bacterial motility and organize multicellular behavior. ISME J.

[7] Lux R, Li Y, Lu A, Shi W (2004). Detailed three-dimensional analysis of structural features of *Myxococcus xanthus* fruiting bodies using confocal laser scanning microscopy. Biofilms.

[8] Mauriello EM, Mignot T, Yang Z, Zusman DR (2010). Gliding motility revisited: how do the myxobacteria move without flagella?. Microbiol Mol Biol Rev.

[9] Spormann AM (1999). Gliding motility in bacteria: insights from studies of *Myxococcus xanthus*. Microbiol Mol Biol Rev.

[10] Youderian P, Hartzell PL (2006). Transposon insertions of magellan-4 that impair social gliding motility in *Myxococcus xanthus*. Genetics.

[11] Youderian P, Burke N, White DJ, Hartzell PL (2003). Identification of genes required for adventurous gliding motility in *Myxococcus xanthus* with the transposable element mariner. Molecular microbiology.

[12] Faure LM (2016). The mechanism of force transmission at bacterial focal adhesion complexes. Nature.

[13] Nan B, McBride MJ, Chen J, Zusman DR, Oster G (2014). Bacteria that glide with helical tracks. Curr Biol.

[14] Balagam R (2014). *Myxococcus xanthus* gliding motors are elastically coupled to the substrate as predicted by the focal adhesion model of gliding motility. PLoS Comput Biol.

[15] Mignot T, Shaevitz JW, Hartzell PL, Zusman DR (2007). Evidence that focal adhesion complexes power bacterial gliding motility. Science.

[16] Chang YW (2016). Architecture of the type IVa pilus machine. Science.

[17] Wu SS, Kaiser D (1995). Genetic and functional evidence that Type IV pili are required for social gliding motility in *Myxococcus xanthus*. Mol Microbiol.

[18] Wu SS, Wu J, Kaiser D (1997). The *Myxococcus xanthus pilT* locus is required for social gliding motility although pili are still produced. Mol Microbiol.

[19] Clausen M, Jakovljevic V, Sogaard-Andersen L, Maier B (2009). High-force generation is a conserved property of type IV pilus systems. J Bacteriol.

[20] Kaiser D (1979). Social gliding is correlated with the presence of pili in *Myxococcus xanthus*. Proceedings of the National Academy of Sciences of the United States of America.

[21] Jakovljevic V, Leonardy S, Hoppert M, Sogaard-Andersen L (2008). PilB and PilT are ATPases acting antagonistically in type IV pilus function in *Myxococcus xanthus*. J Bacteriol.

[22] Merz AJ, So M, Sheetz MP (2000). Pilus retraction powers bacterial twitching motility. Nature.

[23] Skerker JM, Berg HC (2001). Direct observation of extension and retraction of type IV pili. Proc Natl Acad Sci USA.

[24] Yang, Z., Li, C. Y., Friedrich, C. & Sogaard-Andersen, L. (eds Z. Yang & P. I. Higgs) 183–198 (Caister Academic Press, 2014).

[25] Giltner CL, Nguyen Y, Burrows LL (2012). Type IV pilin proteins: versatile molecular modules. Microbiol Mol Biol Rev.

[26] Li Y (2003). Extracellular polysaccharides mediate pilus retraction during social motility of *Myxococcus xanthus*. Proc Natl Acad Sci USA.

[27] Yang Z, Geng Y, Xu D, Kaplan HB, Shi W (1998). A new set of chemotaxis homologues is essential for *Myxococcus xanthus* social motility. Mol. Microbiol..

[28] Zhou T, Nan B (2017). Exopolysaccharides promote *Myxococcus xanthus* social motility by inhibiting cellular reversals. Mol Microbiol.

[29] Black WP, Yang Z (2004). *Myxococcus xanthus* chemotaxis homologs DifD and DifG negatively regulate fibril polysaccharide production. J Bacteriol.

[30] Black WP, Wang L, Davis MY, Yang Z (2015). The orphan response regulator EpsW is a substrate of the DifE kinase and it regulates exopolysaccharide *in Myxococcus xanthus*. Sci Rep.

[31] Black WP, Xu Q, Yang Z (2006). Type IV pili function upstream of the Dif chemotaxis pathway in *Myxococcus xanthus* EPS regulation. Mol Microbiol.

[32] Black WP, Schubot FD, Li Z, Yang Z (2010). Phosphorylation and dephosphorylation among Dif chemosensory proteins essential for exopolysaccharide regulation in *Myxococcus xanthus*. J Bacteriol.

[33] Yang Z, Li Z (2005). Demonstration of interactions among *Myxococcus xanthus* Dif chemotaxis-like proteins by the yeast two-hybrid system. Archives of microbiology.

[34] Weimer RM, Creighton C, Stassinopoulos A, Youderian P, Hartzell PL (1998). A chaperone in the HSP70 family controls production of extracellular fibrils in *Myxococcus xanthus*. J Bacteriol.

[35] Dana JR, Shimkets LJ (1993). Regulation of cohesion-dependent cell interactions in *Myxococcus xanthus*. J Bacteriol.

[36] Moak PL, Black WP, Wallace RA, Li Z, Yang Z (2015). The Hsp70-like StkA functions between T4P and Dif signaling proteins as a negative regulator of exopolysaccharide in *Myxococcus xanthus*. PeerJ.

[37] Lu A (2005). Exopolysaccharide biosynthesis genes required for social motility in *Myxococcus xanthus*. Molecular microbiology.

[38] Wall D, Kaiser D (1999). Type IV pili and cell motility. Mol Microbiol.

[39] Black, W. P. *et al*. Isolation and characterization of a suppressor mutation that restores Myxococcus xanthus exopolysaccharide production. Microbiology 155, 3599–3610, doi:10.1099/mic.0.031070-0 (2009). 10.1099/mic.0.031070-0 PMC2879065 19684067

[40] Schaaper RM, Cornacchio R (1992). An *Escherichia coli dnaE* mutation with suppressor activity toward mutator *mutD5*. J Bacteriol.

[41] Vlamakis HC, Kirby JR, Zusman DR (2004). The Che4 pathway of *Myxococcus xanthus* regulates type IV pilus-mediated motility. Mol Microbiol.

[42] Wallace RA, Black WP, Yang X, Yang Z (2014). A CRISPR with roles in *Myxococcus xanthus* development and exopolysaccharide production. Journal of bacteriology.

[43] Mancl JM, Black WP, Robinson H, Yang Z, Schubot FD (2016). Crystal Structure of a Type IV Pilus Assembly ATPase: Insights into the Molecular Mechanism of PilB from *Thermus thermophilus*. Structure.

[44] Li C, Wallace RA, Black WP, Li YZ, Yang Z (2013). Type IV pilus proteins form an integrated structure extending from the cytoplasm to the outer membrane. PLoS One.

[45] McCallum M, Tammam S, Khan A, Burrows LL, Howell PL (2017). The molecular mechanism of the type IVa pilus motors. Nature communications.

[46] Reindl S (2013). Insights into FlaI functions in archaeal motor assembly and motility from structures, conformations, and genetics. Mol Cell.

[47] Wendler P, Ciniawsky S, Kock M, Kube S (2012). Structure and function of the AAA + nucleotide binding pocket. Biochim Biophys Acta.

[48] Erzberger JP, Berger JM (2006). Evolutionary relationships and structural mechanisms of AAA+ proteins. Annu Rev Biophys Biomol Struct.

[49] Black, W. P. *et al*. Isolation and characterization of a suppressor mutation that restores *Myxococcus xanthus* exopolysaccharide production. *Microbiology* (2009). 10.1099/mic.0.031070-0 PMC2879065 19684067

[50] Rose I (2011). Identification and characterization of a unique, zinc-containing transport ATPase essential for natural transformation in *Thermus thermophilus* HB27. Extremophiles.

[51] Niesen FH, Berglund H, Vedadi M (2007). The use of differential scanning fluorimetry to detect ligand interactions that promote protein stability. Nature protocols.

[52] Senisterra GA, Finerty PJ (2009). High throughput methods of assessing protein stability and aggregation. Mol Biosyst.

[53] Yamagata A, Tainer JA (2007). Hexameric structures of the archaeal secretion ATPase GspE and implications for a universal secretion mechanism. Embo J.

[54] Yount RG, Ojala D, Babcock D (1971). Interaction of P-N-P and P-C-P analogs of adenosine triphosphate with heavy meromyosin, myosin, and actomyosin. Biochemistry.

[55] Kelly SM, Jess TJ, Price NC (2005). How to study proteins by circular dichroism. Biochim Biophys Acta.

[56] Jameson DM, Eccleston JF (1997). Fluorescent nucleotide analogs: synthesis and applications. Methods Enzymol.

[57] Chen X (2010). An ATPase promotes autophosphorylation of the pattern recognition receptor XA21 and inhibits XA21-mediated immunity. Proc Natl Acad Sci USA.

[58] Matchkov VV, Krivoi II (2016). Specialized Functional Diversity and Interactions of the Na,K-ATPase. Front Physiol.

[59] Liu J, Xie ZJ (2010). The sodium pump and cardiotonic steroids-induced signal transduction protein kinases and calcium-signaling microdomain in regulation of transporter trafficking. Biochim Biophys Acta.

[60] Haas M, Wang H, Tian J, Xie Z (2002). Src-mediated inter-receptor cross-talk between the Na+/K+ -ATPase and the epidermal growth factor receptor relays the signal from ouabain to mitogen-activated protein kinases. J Biol Chem.

[61] Haas M, Askari A, Xie Z (2000). Involvement of Src and epidermal growth factor receptor in the signal-transducing function of Na+/K+ -ATPase. J Biol Chem.

[62] Cherfils J, Zeghouf M (2011). Chronicles of the GTPase switch. Nature chemical biology.

[63] Cherfils J, Zeghouf M (2013). Regulation of small GTPases by GEFs, GAPs, and GDIs. Physiological reviews.

[64] Hengge R (2009). Principles of c-di-GMP signalling in bacteria. Nat Rev Microbiol.

[65] Sondermann H, Shikuma NJ, Yildiz FH (2012). You’ve come a long way: c-di-GMP signaling. Curr Opin Microbiol.

[66] Hengge R, Grundling A, Jenal U, Ryan R, Yildiz F (2016). Bacterial Signal Transduction by Cyclic Di-GMP and Other Nucleotide Second Messengers. J Bacteriol.

[67] Conner JG, Zamorano-Sanchez D, Park JH, Sondermann H, Yildiz FH (2017). The ins and outs of cyclic di-GMP signaling in *Vibrio cholerae*. Curr Opin Microbiol.

[68] Wang YC (2016). Nucleotide binding by the widespread high-affinity cyclic di-GMP receptor MshEN domain. Nature communications.

[69] Jones CJ (2015). C-di-GMP Regulates Motile to Sessile Transition by Modulating MshA Pili Biogenesis and Near-Surface Motility Behavior in *Vibrio cholerae*. PLoS Pathog.

[70] Roelofs KG (2015). Systematic Identification of Cyclic-di-GMP Binding Proteins in *Vibrio cholerae* Reveals a Novel Class of Cyclic-di-GMP-Binding ATPases Associated with Type II Secretion Systems. PLoS Pathog.

[71] Hendrick WA, Orr MW, Murray SR, Lee VT, Melville SB (2017). Cyclic-di-GMP Binding by an Assembly ATPase (PilB2) and Control of Type IV Pilin Polymerization in the Gram-positive Pathogen *Clostridium perfringens*. J Bacteriol.

[72] Skotnicka D (2015). Cyclic Di-GMP Regulates Type IV Pilus-Dependent Motility in *Myxococcus xanthus*. J Bacteriol.

[73] Skotnicka D (2016). A Minimal Threshold of c-di-GMP Is Essential for Fruiting Body Formation and Sporulation in *Myxococcus xanthus*. PLoS Genet.

[74] Campos JM, Zusman DR (1975). Regulation of development in *Myxococcus xanthus*: effect of 3′:5′-cyclic AMP, ADP, and nutrition. Proceedings of the National Academy of Sciences of the United States of America.

[75] Miller, J. H. *Experiments in molecular genetics* (Cold Spring Harbor Laboratory, 1972).

[76] Magrini V, Creighton C, Youderian P (1999). Site-specific recombination of temperate *Myxococcus xanthus* phage Mx8: genetic elements required for integration. J Bacteriol.

[77] Julien B, Kaiser AD, Garza A (2000). Spatial control of cell differentiation in *Myxococcus xanthus*. Proceedings of the National Academy of Sciences of the United States of America.

[78] Li SF, Shimkets LJ (1988). Site-specific integration and expression of a developmental promoter in *Myxococcus xanthus*. J Bacteriol.

[79] Wall D, Kolenbrander PE, Kaiser D (1999). The *Myxococcus xanthus pilQ (sglA)* gene encodes a secretin homolog required for type IV pilus biogenesis, social motility, and development. J Bacteriol.

[80] Kashefi K, Hartzell PL (1995). Genetic suppression and phenotypic masking of a *Myxococcus xanthus frzF*- defect. Molecular microbiology.

[81] Ueki T, Inouye S, Inouye M (1996). Positive-negative KG cassettes for construction of multi-gene deletions using a single drug marker. Gene.

