# Supplementary material for: The type IV pilus assembly ATPase PilB functions as a signaling protein to regulate exopolysaccharide production in Myxococcus xanthus
Source: Sci Rep. 2017 Aug 4;7:7263. doi: 10.1038/s41598-017-07594-x (PMC5544727; doi:10.1038/s41598-017-07594-x)
Supplement: Supplementary file 1 — Supplemental Information [file 41598_2017_7594_MOESM1_ESM.pdf]

Supplemental Information

The type IV pilus assembly ATPase PilB functions in a signal transduction pathway to regulate exopolysaccharide production in *Myxococcus xanthus*

Wesley P. Black, Lingling Wang, Xing Jing, Rafael Castañeda Saldaña, Feng Li, Birgit E. Scharf, Florian D. Schubot and Zhaomin Yang[1]

Department of Biological Sciences, Virginia Polytechnic Institute and State University, Blacksburg, VA 24061

[1]To whom correspondence should be addressed. E-mail: zmyang@vt.edu.

Running title: An ATPase in biofilm regulation

Key word: Biofilm; c-di-GMP, EPS, Motility, Signaling ATPase

SUPPLEMENTAL MATERIALS AND METHODS

Protein purification. For the heterologous expression of PilB in *E. coli*, the coding region for the equivalent of *M. xanthus* PilB from *T. thermophilus* HB27 as shown in Fig. S4 [1,2] was PCR amplified and cloned into the BamHI and HindIII sites of pQE30 (Qiagen). The resulting plasmid pWB750 expresses $PilB^{WT}$ with an N-terminal 6×His tag. pWB751 and pWB752 were constructed using pWB750 as a template by a two-step overlap PCR using mutagenic primers to express $PilB^{WA}$ and $PilB^{WB}$ (Fig. S4).

Induced cells were harvested by centrifugation at 5,000 × g for 15 minutes and stored at -80°C until needed. Cell pellets were resuspended in 30 ml of buffer A (25 mM Tris-Cl, 500 mM NaCl, 25 mM imidazole, 10% glycerol, pH 7.8) containing 2.5 mM 2-mercaptoethanol, DNase I (5 µg/ml) and the recommended amount of Protease Inhibitor Cocktail VII (Research Products International). Cells were lysed by two passes at 18,000 p.s.i. using a French Press (Thermo Scientific), cleared of cell debris by centrifugation at 12,000 × g for 30 minutes followed by one hour at 100,000 × g. The supernatant was heated at 65°C for 30 min, cooled on ice for 15 min and precipitated proteins were removed by centrifugation at 12,000 × g for 30 minutes.

The resulting supernatant above was passed through a 0.45 µm filter prior to loading on a 5 ml HisTrap FF (GE Healthcare) column equilibrated with buffer A. Protein purification was performed by fast protein liquid chromatography (FPLC) using an ÄKTA Prime (GE Healthcare). The column was washed until the initial baseline A_{280} was restored, typically 10-15 column volumes of buffer A. Proteins were eluted from the column using a 25-500 mM linear imidazole gradient equivalent to 20 column volumes (100 ml). Elution fractions with peak A_{280} readings were collected and analyzed by SDS-PAGE. Fractions of interest were concentrated and

applied to a Superdex 200 prep grade 10/300 column (GE Healthcare) equilibrated with storage buffer (25 mM Tris-Cl [pH 7.8], 150 mM NaCl and 10% glycerol) for further fractionation by size exclusion chromatography (SEC). Fractions containing PilB at over 95% purity as analyzed by SDS-PAGE analysis were concentrated in storage buffer using Amicon stirred cells with 30 kD cut-off membranes (Millipore). Protein concentrations were measured using the Bio-Rad Protein Assay, adjusted to 1 mg/ml or about 15.6 µM, aliquoted and stored at -80°C until use.

REFERENCES

1 Rose, I. *et al.* Identification and characterization of a unique, zinc-containing transport ATPase essential for natural transformation in *Thermus thermophilus* HB27. *Extremophiles*, doi:10.1007/s00792-010-0343-2 (2011).

2 Jakovljevic, V., Leonardy, S., Hoppert, M. & Sogaard-Andersen, L. PilB and PilT are ATPases acting antagonistically in type IV pilus function in *Myxococcus xanthus*. *J Bacteriol* **190**, 2411-2421, doi:10.1128/JB.01793-07 (2008).

3 Wall, D. & Kaiser, D. Type IV pili and cell motility. *Mol. Microbiol.* **32**, 1-10 (1999).

4 Goldman, B. S. *et al.* Evolution of sensory complexity recorded in a myxobacterial genome. *Proc Natl Acad Sci USA* **103**, 15200-15205 (2006).

5 Wang, Y. C. *et al.* Nucleotide binding by the widespread high-affinity cyclic di-GMP receptor MshEN domain. *Nature communications* **7**, 1-12, doi:10.1038/ncomms12481 (2016).

SUPPLEMENTAL FIGURES

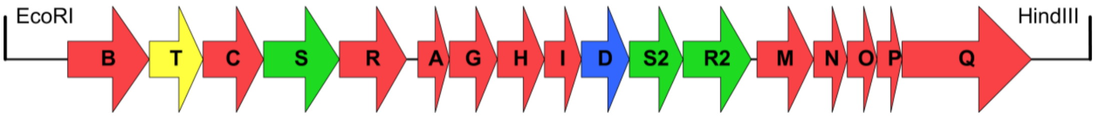

Fig. S1. *Myxococcus xanthus pil* locus [3,4]. The *pil* region shown between EcoRI and HindIII is 23.0 kb. The filled arrows indicate the coding regions of *pil* genes to scale. Single letters designate different *pil* genes. The *pilBTC*, *pilGHI* and *pilMNOPQ* gene clusters were targeted for mutagenesis in this study.

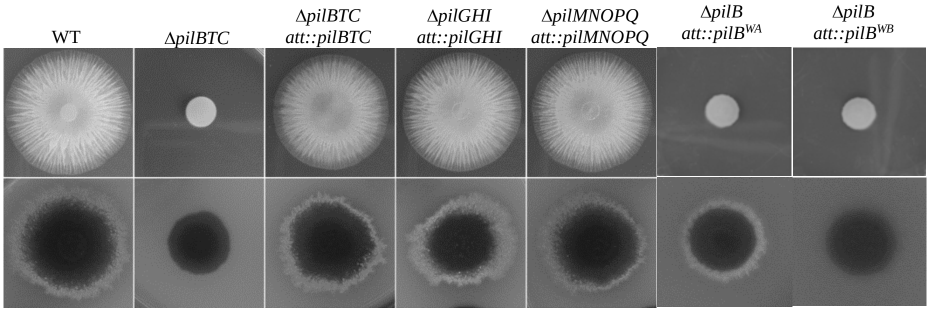

Fig. S2. Complementation of deletions of *pil* gene clusters. S motility (upper) and EPS production (lower) were examined on plates with 0.4% agar and those with Calcofluor white, respectively. Strains: DK1622 (WT), YZ1636 (Δ*pilBTC*), YZ1644 (Δ*pilBTC att*::*pilBTC*), YZ1870 (Δ*pilGHI att*::*pilGHI*) and YZ1645 (Δ*pilMNOPQ att*::*pilMNOPQ*). YZ1865 (Δ*pilGHI*) and YZ1635 (Δ*pilMNOPQ*) showed the same phenotype as YZ1636 (data not shown)

```
MxPilB ------MSGRLGELLVRENLISVQQLRKAQEEQQKNGTRIGTALVKTGAIEESKLTDFLSKQYGVPAINL
TtPilB MQKGLPRAKPLGEILVELGLARPEDVEEALQKQRRGGGRLEDTLVQSGKLRPEALAQAVATQLGYPYVDP

MxPilB KDFDVEPDIIKLVPKEVAEKHLVVPVNRAGPSLIVAMCDPSNIFAVDDLKFL-----TGYNIETVVASEV
TtPilB EEDPPDPGAPLLLPEDLCRRYGVFPHRLEGNRLVLLMKDPRNILALDDVRLALKRKGLNYEVAPAVATEA

MxPilB SIREAIERYYAEKGPSLEDIVGDVGDDIEVTKEETENIDEMAKAADDAPVVKLVNLILMDAIKKRASDIH
TtPilB AITKLIERFYGK--AELSEIAK------EFAKKQAEEEVPSPLELDESAAQKFVKQVIREAFLQDASDIH

MxPilB VEPYEKDFRVRFRIDGVMYEVMRPPMKLRNAITSRLKIMASLDISERRLPQDGRIKIKMGGGKEMDFRVS
TtPilB IEPRQNDVQVRLRIDGALRPYSTLPKGALNAVISVVKIMGGLNIAEKRLPQDGRVRYR-EGAIDVDLRLS
                                                              Walker A
MxPilB VCPTLFGEKVVMRLLDKSNLQLDMTKLGFDAQPLAWFKEAIDRPYGMVLVTGPTGSGKTTTLYSALSSLN
TtPilB TLPTVYGEKAVMRLLKKASDIPEIEDLGFAPGVFERFKEVISKPYGIFLITGPTGSGKSFTTFSILKRIA
                                                    Walker B
MxPilB GLDTNICTAEDPVEFNFAGINQVQMHDDIGLNFAAALRSFLRQDPDIIMIGEIRDFETAEIGVKAALTGH
TtPilB TPDKNTQTIEDPVEYEIPGINQTQVNPQAGLTFARALRAFLRQDPDIIMVGEIRDSETAKIATEAALTGH

MxPilB LVLSTLHTNDAPGTVSRLLNMGIEPFLVTASLNLILAQRLARRLCPACKKPAENVDEQALIDAGVPPDKI
TtPilB LVIATLHTNDAAQAITRLDEMGVEPFNISAALIGVLSQRLVRRVCEHCKVEVKP-DPETLRRLGLSEAEI

MxPilB GTFTMYEKVGCRDCNDRGYRGRVAIYEVMPFWDGLKELVINGASAAELKQEAIRLGMSSLRMSGLRKMMD
TtPilB QGARLYKGMGCERCGGTGYKGRYAIHELLVVDDEIRHAIVAGKSATEIKEIARRKGMKTLREDGLYKALQ

MxPilB GATTLEEVVGNTAPDRF
TtPilB GITTLEEVLARTIE---
```

A

```
VcMshE   LRKRLGDLLVEEGIVSEAQLEQALNAQKNTGRRLGDTLISLGFLSETQLLNFLAQQ
MxPilB   MSGRLGELLVRENLISVQQLRKAQEEQQKNGTRIGTALVKTGAIEESKLTDFLSKQ
```

B

Fig.S3. A. Alignment of *M. xanthus* (Mx) PilB and with the region of *T. thermophilus* (Tt) PilB [1,2] used in this study. Highlighted in aqua are identical residues and in gray are conserved ones. Walker A (WA) and Walker B (WB) residues are boxed for both proteins [1,2]. Highlighted in red is M388 that was mutated to an Ile in MxPilB*. Highlighted in yellow are the strictly conserved Lys (K) and Glu (E) that were substituted by Ala in $PilB^{WA}$ and $PilB^{WB}$, respectively. **B.** c-di-GMP binding motifs in MxPilB (underlined in **A**) are aligned with residues 6-61 of *Vibrio cholerae* (Vc*)* MshE. There are two tandem c-di-GMP binding motifs in VcMshE. Each is 24-residues long and binds half of the c-di-GMP molecule [5]. Highlighted in green and turquoise are the consensus for the two motifs in VcMshE, respectively. The residues in MxPilB are similarly colored with yellow indicating conserved changes.

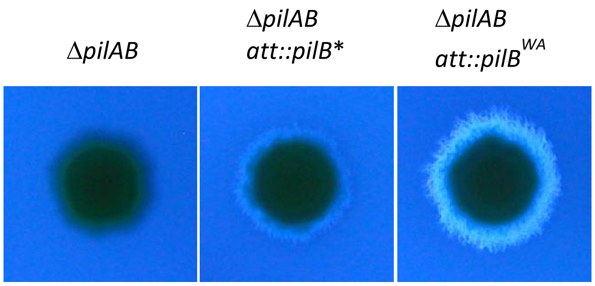

Fig S4. Examination of EPS production on media containing Calcofluor white. Strains: YZ1682 (Δ*pilAB*), YZ1650 (Δ*pilAB att*::*pilB**) and YZ1504 (Δ*pilAB att*::*pilB*^{WA}).

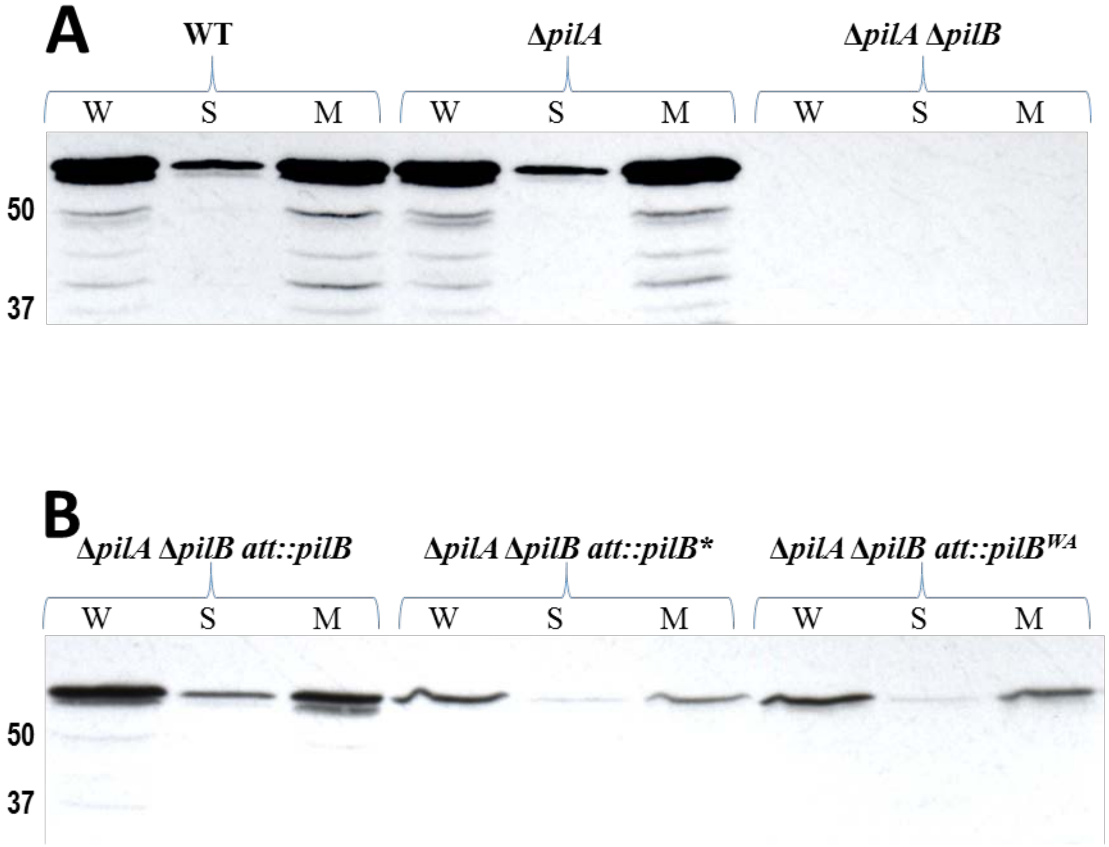

Fig. S5. Examination of PilB expression by immunoblotting. Panel **A**. DK1622 (WT), YZ690 (Δ*pil*A), YZ1682 (Δ*pilA* Δ*pilB*). Panel **B**: YZ1849 (Δ*pilA* Δ*pilB att::pilB*), YZ1850 (Δ*pilA* Δ*pilB att::pilB**), YZ1504 (Δ*pilA* Δ*pilB att::pilB*^{WA}). Cells resuspended in lysis buffer (10 mM HEPES, 50 mM NaCl, 4 mM PMSF, pH= 7.2) were sonicated and cell debris was then removed by centrifugation at 15,000×g for 15 minutes to produce the whole cell lysate (W). Membrane (M) and Soluble (S) fractions were pellets and supernatants from the whole cell lysate after centrifugation at 160,000×g for 1.5 hours. Samples from 5×10^{7} were loaded on each lane. The outer edges of the original immunoblots were cropped out.

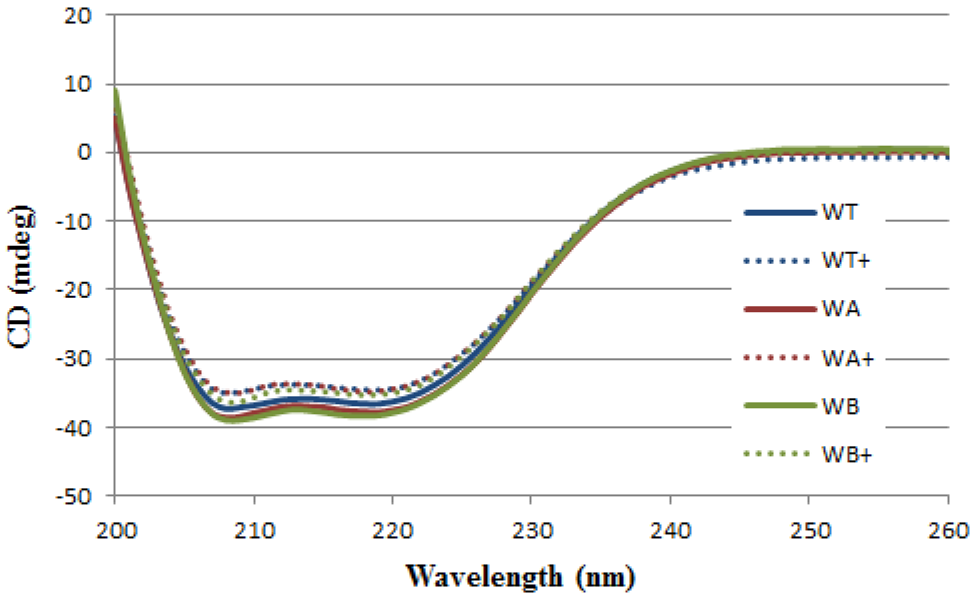

Fig. S6. Examination of PilB proteins by far UV circular dichroism (see SI Materials and Methods for details). WT stands for $PilB^{WT}$, WA for $PilB^{WA}$ and WB for $PilB^{WB}$. The plus sign (+) indicates supplementation of 0.1 mM ATP. The addition of 0.1 mM ADP had no effect on the spectrum of these proteins (data not shown).
